# What might working from home mean for the geography of work and commuting in the Greater Golden Horseshoe, Canada?

**DOI:** 10.1177/00420980231186499

**Published:** 2023-08-07

**Authors:** Matthias Sweet, Darren M Scott

**Affiliations:** Toronto Metropolitan University, Canada; McMaster University, Canada

**Keywords:** commuting, downtown, telework, urban geography, working from home, 通勤, 市中心, 远程办公, 城市地理, 在家办公

## Abstract

The Covid-19 pandemic has highlighted the precarity of urban society, illustrating both opportunities and challenges. Teleworking rates increased dramatically during the pandemic and may be sustained over the long term. For transportation planners, these changes belie the broader questions of how the geography of work and commuting will change based on pandemic-induced shifts in teleworking and what this will mean for society and policymaking. This study focuses on these questions by using survey data (*n* = 2580) gathered in the autumn of 2021 to explore the geography of current and prospective telework. The study focuses on the Greater Golden Horseshoe, the mega-region in Southern Ontario, representing a fifth of Canadians. Survey data document telework practices before and during the pandemic, including prospective future telework practices. Inferential models are used to develop working-from-home scenarios which are allocated spatially based on respondents’ locations of work and residence. Findings indicate that telework appears to be poised to increase most relative to pre-pandemic levels around downtown Toronto based on locations of work, but increases in teleworking are more dispersed based on employees’ locations of residence. Contrary to expectations by many, teleworking is not significantly linked to home–work disconnect – suggesting that telework is poised to weaken the commute–housing trade-off embedded in bid rent theory. Together, these results portend a poor outlook for downtown urban agglomeration economies but also more nuanced impacts than simply inducing sprawl.

## 摘要

新冠疫情凸显了城市社会的不稳定性，表明了机遇与挑战并存。在新冠疫情期间，远程工作率急剧上升，并可能长期保持下去。对于交通规划者来说，这些变化掩盖了更广泛的问题，即由于疫情引起的远程办公的转变，工作的地理位置和通勤会如何变化，以及这对社会和政策制定将意味着什么。本研究利用**2021** 年秋季收集的调查数据（**n= 2580**），重点考察了这些问题，以探索当前和未来远程工作的地理分布情况。本文重点研究了大金马蹄地区（**Greater Golden Horseshoe**），这是安大略省南部的特大地区，占加拿大人口的五分之一。调查数据记录了新冠疫情之前和期间的远程工作实践，包括预期的未来远程工作实践。推理模型用于制定在家办公方案，并根据受访者的工作地点和居住地点进行空间分配。研究结果表明，根据工作地点来看，相对于新冠疫情前的水平，多伦多市中心附近的远程工作似乎增加最多，但根据员工的居住地点来看，远程工作的增加则较为分散。与许多人的预期相反，远程办公与家与工作脱离之间的关系并不显著--这表明远程办公有望削弱竞租理论中的通勤与住房权衡。总之，这些结果预示着市中心城市集聚经济的前景不佳，但是远程办公可能会带来更微妙的影响而不仅仅是导致无序扩张。

## Introduction

Up to a third of jobs in developed economies may be completed from home, leading to expectations of more telework after the Covid-19 pandemic than before (Moens et al., 2022). But the broader social impacts are unclear. While many governments have been reluctant (Hynes, 2014) or slow (Stiles, 2020) to manage teleworking, policymakers (Bachelet et al., 2021; Samek Lodovici et al., 2021) are frantically trying to learn how to address future telework. Telework policies have emerged chiefly from idiosyncratic transportation policy paths (Stiles, 2020) or from common labour markets (e.g. the European Union) (Welz and Wolf, 2010), so the need to understand the role of telework in a broader social context is growing.

Teleworking only started growing significantly in the dozen years leading up to the pandemic (Stiles and Smart, 2021). Since the pandemic started, telework has increased precipitously and appears likely to remain higher than before (Sweet and Scott, 2022). Many potential benefits from telework have been touted, including energy savings (Villeneuve et al., 2021), congestion alleviation (Nguyen, 2021) and real estate cost savings (Ruth and Chaudhry, 2008). But longer-term impacts on cities are also possible, including urban spatial restructuring, local fiscal pressure, inter-jurisdictional fiscal competition and weaker unions (Brueckner et al., 2021). Of the potential implications, one critical policy question for planners and geographers is what the future of telework might mean for urban spatial structure and the geography of how cities are organised and function. Shearmur (2021) posits that work location should be understood more as a ‘probability space’ than a single place of work – making the geography of work multidimensional and complicated. This study aims to help address this issue and focuses on the question of how telework may influence the geography of work and commuting.

## Literature review

Telework represents a significant opportunity to curb transportation-induced greenhouse gas emissions, alleviate congestion and reduce energy use (Bachelet et al., 2021; Hook et al., 2020), but its uptake could accelerate suburban sprawl (Larson and Zhao, 2017). Towards understanding how telework affects spatial structure, this study first discusses the literature on telework adoption and travel behaviour implications. It then explores the implications of telework for urban spatial structure by highlighting lessons from bid rent theory, from generalised equilibrium models and from empirical studies. Each of these literatures has their own advantages and disadvantages, so they must be considered in conjunction when framing this study and exploring the links between telework and spatial structure in the aftermath of the pandemic.

### Telework adoption

Early initiatives to encourage teleworking were slow due to uncertain advantages and clear downsides to telework (Aguilera et al., 2016). Growth in uptake has only accelerated in the last two decades (Stiles and Smart, 2021), partly due to complex socialisation processes (Scott et al., 2012; Wilton et al., 2011). Estimating telework uptake is particularly challenging because most standard travel or employment surveys are not well designed for the complexity of remote or hybrid work (Shearmur, 2021). Despite early optimism that telework could decrease travel demand (Pendyala et al., 1992), this promise has largely fallen flat (Guerin, 2021; Salomon and Mokhtarian, 2007). Instead, information and communication technologies (ICT) typically complement (rather than substitute for) travel (Mokhtarian, 2003; Zhu et al., 2018). While nomadic work (in non-traditional locations) and overtime work from home have accelerated rapidly and complicated the geography of work, home-based telework has only recently grown (Aguilera et al., 2016). Presciently, Aguilera et al. (2016: 10) warn that telework is likely to remain a small, niche choice, ‘without an exogenous shock’. The Covid-19 pandemic may have represented precisely such an exogenous shock which has shifted both individual workers and entire work processes involving networks of people to be completed remotely (Moens et al., 2022).

Policymakers are still unclear how to respond to telework uptake (Zenkteler et al., 2022). Policy efforts to promote telework have existed at least since the 1970s in the USA (Stiles, 2020) and the 1990s in Canada (Bussiere and Lewis, 2002). Telework has been most common among knowledge-sector workers, managers, individuals with job flexibility (Stiles and Smart, 2021) and more highly-educated individuals (Sweet and Scott, 2022). Nevertheless, institutional barriers to expanding teleworking include the dominance of existing work, automobility, a dearth of supportive policy and a perceived lack of legitimacy (Hynes, 2016).

Newer research explores what pandemic-induced growth in telework may mean for broader social outcomes, including firm locations (Smailes et al., 2021), energy use (O’Brien and Yazdani Aliabadi, 2020) and public transit (Morissette et al., 2021). Private teleworking benefits appear to be approximately US$10,000–20,000 annually (Hambly and Lee, 2019). Teleworking benefits are also uncertain: it may (Tavares, 2017) or may not improve individual workers’ health (Mahmoudi and Zhang, 2022), and it may impact affective wellbeing (Anderson et al., 2015). It is especially unclear whether teleworkers’ private welfare improvements offset broader social and environmental impacts, including sprawl (Moos et al., 2006), leading Behrens et al. (2021) to caution that high rates of teleworking may be ‘too much of a good thing’.

### Telework and travel

While some studies of telework and travel simply focus on telework and commuting, others explore how telework shapes other travel behaviour (including non-commuting trips). First, studies have confirmed the potential for telework to reduce commuting-related travel times and vehicle use (Giovanis, 2018). While work and home may be more disconnected for teleworkers, lower commuting frequencies generally offset higher distances (Zhu, 2013). Despite these associative links, causation is murkier. It is unclear whether individuals choose more disconnected work–home arrangements in anticipation of teleworking (implying that telework induces ‘sprawl’) or whether teleworking becomes an adaptation strategy for individuals with longer commutes (implying that telework offsets commuting-related travel). Ory and Mokhtarian (2006) focus on a small sample of California commuters, finding more evidence of the latter (telework offsetting commuting) than the former – implying that teleworking (at least in the early 2000s) appeared not to have magnified the work–home disconnect. Regardless, insofar that an increased job–housing disconnect and telework appear to be complementary and to support the ‘dominant postulate’ of the land–commuting trade-off (Moos and Skaburskis, 2008), the direction of causation may be only of secondary importance with respect to policy implications.

Second, more comprehensive studies have focused on whether telework may attenuate overall travel demand (Mokhtarian et al., 2006). There is strong evidence that ICT and telework may induce additional discretionary travel, leading telework to negligibly impact overall travel (Caldarola and Sorrell, 2022; Cerqueira et al., 2020; Wöhner, 2022) and maybe even to induce more travel (He and Hu, 2015; Ravalet and Rérat, 2019; Zhu and Mason, 2014). Several nuances on the link between telework and travel emerge. For example, Caldarola and Sorrell (2022) find telework–travel threshold effects above or below which telework decreases net travel. Cerqueira et al. (2020) contend that the potential for telework to offset travel demand may be less promising in European cities (where existing transit users are most likely to switch to telework) than in North America (where vehicle use is higher).

However, studies using a time use perspective or those focusing on the Covid-19 aftermath are more optimistic about telework reducing travel demand. According to Bieser et al. (2022), telework and commuting time savings lead to more time engaging in more enjoyable non-travel activities. Elldér (2020) finds potential for telework to reduce travel demand, noting that it should be normatively interpreted as one of many time use coping strategies, rather than as being a travel complement or substitute. Telework can reduce overall travel (Hensher et al., 2021a; Rafiq et al., 2022), and such reductions may continue over the long term (Hensher et al., 2021a, 2021b). Overall, telework appears poised to shape the geography of discretionary travel, shifting it away from work and towards individuals’ homes.

### Telework and spatial structure

Telework is expected to affect spatial structure and may stand to weaken key assumptions in urban economics. According to the bid rent model, firms and residents bid on land based on trade-offs between demand for land and generalised travel costs (Weinberger, 2007). This model has several key assumptions, three of which deserve particular attention in light of their potential to be affected by telework.

First, the common single-region model assumes that ‘places of work’ are identifiable, fixed destinations (Shearmur, 2021), leading agglomeration benefits to be both measured and accrued through physical proximity (Circella and Mokhtarian, 2017). This leads to questions about the conditions under which agglomeration benefits in a world with less clear ‘places of work’ may accrue and affect the real estate market. For example, Circella and Mokhtarian (2017) expect more teleworking to induce polycentric sub-centres and regionally scaled agglomeration to be more important agglomerative forces than the physical spaces of traditional downtowns.

Second, insofar as telework affects commuting behaviour, it is expected to change spatial structure through its impact on generalised travel costs but is likewise poised to reduce the impact of generalised travel costs on spatial structure. On the one hand, one may imagine less commuting to lead to marginally less road congestion (Loo and Huang, 2022), which (in turn) may induce further work–housing disconnect (Sweet, 2011). On the other hand, less transit commuting may affect farebox recovery ratios, further degrade transit services, increase transit travel costs and hence induce mode switching (Loa et al., 2021; Parker et al., 2021) and/or firm residential relocations (Mashrur et al., 2022). But should telework feature as a choice which is divorced from land–commuting trade-offs, this stands to significantly upend how city-regions are organised.

Third, the bid rent model assumes that wage rates and real estate markets in other city-regions are unimportant because individuals reside, commute and work in the same city-region. However, as telework could lead individuals to seek housing in different cities (Moos and Skaburskis, 2010), this necessitates bid rent theory to consider systems of cities (Gokan et al., 2021), leading to the potential need to revisit the nature of postmodern megaregions.

Two very different bodies of literature inform knowledge on the links between telework and urban spatial structure: (1) simulations of actors in a general equilibrium urban economic model and (2) empirical evidence using imperfect and non-experimental real-world observations. While findings from simulations provide more clarity, they largely ignore context. In contrast, empirical studies are inherently contextual, but they demonstrate how muddy the evidence really is. Each of these lines of inquiry is discussed in turn.

#### Urban economic model simulations

Urban economists’ theoretical work and simulations portend potentially pernicious (Behrens et al., 2021) implications for cities should telework become common (Brueckner et al., 2021). Findings indicate that place-based amenities will become more central to real estate markets as unique differentiators, rather than traditional factors of production, including agglomeration, land, labour and travel costs (Gaigne et al., 2022). Excess teleworking may emerge, should employers or governments not intervene, leading to both allocative and distributive consequences (Kyriakopoulou and Picard, 2021).

As expected based on bid rent theory, telework is likely to increase job–housing disconnect and sprawl (Moeckel, 2017; Rhee, 2009), leading workers to reside up to 75% further from their jobs (Delventhal and Parkhomenko, 2022). Despite job–housing disconnect, regionally scaled geography may still matter for occasional in-person interactions (Moeckel, 2017). Teleworking is expected to lead to a flatter density gradient (Delventhal et al., 2022; Kyriakopoulou and Picard, 2021), larger lots and more interior space (Larson and Zhao, 2017).

In systems of city-regions, teleworking will have broader impacts (Rhee, 2009), including teleworking from smaller, lower-cost cities to jobs in larger, higher-cost cities (Gokan et al., 2021). Widespread telework may make central agglomerations less valuable, leading to an equalisation of real estate prices and wage rates (Behrens et al., 2021; Davis et al., 2022). But while central agglomerations are likely to be less valuable in smaller cities, larger centres may retain significant agglomeration benefits (Delventhal and Parkhomenko, 2022; Kyriakopoulou and Picard, 2021).

#### Empirical evidence

Empirical evidence on telework and urban spatial structure is even muddier. Commercial real estate demand in transit-oriented cities fell partly due to pandemic-induced teleworking (Rosenthal et al., 2022). But telework also reduces peer-to-peer knowledge spillovers, implying downsides (Frakes and Wasserman, 2021). Evidence of teleworking and real estate markets notes that home office availability is gaining importance (Cuerdo-Vilches et al., 2021) and it strengthens home buyers’ preferences for single-family homes (Moos and Skaburskis, 2008). Moos and Skaburskis (2008) caution that small incremental propensities for workers to seek home offices can further engrain norms around single-family homes and sprawl into the housing market. Evidence also suggests that work from home is most prominent in remote areas rather than in big metropolitan areas (Moos and Skaburskis, 2010) – supporting the expectations of inter-city movement of urban economic models (Brueckner et al., 2021; Gokan et al., 2021).

Indirect evidence of telework impacting spatial structure via commuting is highly variant. Public transit users are most willing to telework (Sweet and Scott, 2022), which may lead to fewer transit funds and services and a changing geography of work (Ton et al., 2022). In Tokyo, downtown office workers are most likely to telework, leading Isono and Nara (2022) to expect less transit use, less local travel and longer trip distances. According to de Abreu e Silva (2022), intentions to telework are strongest in suburban areas among individuals with higher commute burdens – implying that telework may simultaneously support suburbanisation and reduce commuting-related travel, as implied in bid rent theory (Moos and Skaburskis, 2010). Others contend that teleworkers’ behavioural motivation may require a rethinking of traditional assumptions around land/travel cost trade-offs in bid rent theory (Elldér, 2017).

Overall, both model simulations and empirical evidence on telework and urban spatial structure provide incomplete guidance. Many suggest that teleworking is likely to lead to sprawl and work–home disconnect but studies continue to struggle with identifying what is likely to unfold with respect to the geography of places of work. Stevens and Shearmur (2020) further question the relevance of traditional location theories based on land–commuting trade-offs considering the complicated spatiality of work. Evidence from others contends that telework may simply map onto and reinforce existing work geographies (Moos and Skaburskis, 2007). Towards providing guidance on these questions, this study explores how the geography of work and commuting is likely to change while considering conventional location theories in the aftermath of pandemic-induced teleworking uptake.

## Research design

Using survey data collected in the autumn of 2021 (*n* = 2580), this study estimates models of teleworking frequency to explore the geography of teleworking before the pandemic, during the pandemic, in the autumn of 2021 and under ‘ideal’ circumstances. Models are estimated based on workers’ places of residence and places of work towards exploring changing geographies of work in the Greater Golden Horseshoe (GGH) of Southern Ontario, Canada. Using inferential models, teleworking market shares are estimated to explore the geography of teleworking. This study defines telework as ‘a workplace arrangement that allows a person to work from home instead of commuting to their usual place of work’.

### Study survey

This study combines data from two separate surveys of respondents aged 18–75 which were explicitly designed in a way that enabled them to be fused together using common survey questions and response options. The Future of Mobility in Canada Survey (henceforth titled the ‘FMCS’) was administered in October and early November 2021 in several major city-regions. Of those respondents, only full-time or part-time employees in the Toronto and Hamilton CMAs are included in this study. Second, the Automated Vehicles in the GGH Survey (henceforth titled the ‘AV Survey’) was administered in late November and early December 2021 to residents of the GGH. The AV Survey data covers a broader geography than that covered by the FMCS (see online Appendix).

Both surveys include respondents’ demographic characteristics, household characteristics, access to mobility tools and travel behaviour and attitudes. The FMCS and AV Survey were respectively administered to panels accessed through Savanta (which contracts with others’ panels) and Dynata (which has its own panel). Both surveys were approved by requisite university ethics boards and typically took 15–20 minutes, and respondents were remunerated using proprietary points programmes.

### Survey weighting

Both surveys used a stratified sampling approach (based on age, sex and geography), but observations are weighted to reflect characteristics of full-time or part-time employed persons aged 18–75 residing in the GGH (see Table 1). Sample weights are estimated using the 2016 Transportation Tomorrow Survey (TTS) (Data Management Group, 2018) based on employees’ places of work and residence. The TTS is chosen as a reference frame due to its common motivation to explore individual, household, spatial structure and travel behaviour outcomes, due to common survey questions and the comprehensiveness of the TTS with which to design weights. Weights are calculated for three occupation types: (1) office (including ‘general office’ and ‘professional, management and technical’), (2) sales and services and (3) manufacturing, construction and trades.

**Table 1.** Survey sample descriptive statistics.

Variable	Characteristic	Sample weighted by place of residence	Sample weighted by place of work	Unweighted SAMPLE	TTS (place of home)	TTS (place of work)
Household size	1	18.16%	18.04%	19.57%	9.48%	9.68%
	2	30.48%	30.51%	29.92%	24.94%	24.99%
	3	22.31%	22.12%	22.36%	22.06%	22.06%
	4+	29.06%	29.33%	28.14%	43.52%	43.26%
Household income (CA$)	< 40,000	9.62%	9.51%	9.92%	9.32%	8.75%
	40,000–99,999	35.85%	35.42%	41.36%	36.26%	36.34%
	100,000+	38.53%	39.40%	41.36%	38.34%	39.29%
	Unknown	16.00%	15.67%	7.36%	16.07%	15.61%
Age group	18–35	26.62%	26.54%	27.95%	35.94%	35.56%
	36–55	49.94%	50.18%	48.14%	47.45%	47.89%
	56–75	23.44%	23.28%	23.91%	16.61%	16.54%
Sex	Female	54.47%	53.99%	50.97%	47.19%	49.23%
	Male	45.53%	46.01%	49.03%	52.81%	50.77%
Student status	Full-time student	5.88%	6.12%	5.39%	4.61%	4.67%
	Part-time student	5.61%	5.78%	5.54%	2.90%	2.89%
	Not a student	88.51%	88.10%	89.07%	92.50%	92.45%
Highest education	No university	22.02%	21.28%	17.83%	Unavailable	
	Some university	65.10%	65.91%	66.98%	Unavailable	
	Graduate or professional degree	12.88%	12.81%	15.19%	Unavailable	
Household vehicle count	0	9.30%	9.03%	9.50%	7.38%	7.60%
	1	42.49%	43.29%	45.93%	27.99%	28.45%
	2	34.54%	34.05%	32.64%	42.13%	42.04%
	3+	13.67%	13.63%	11.94%	22.50%	21.92%
Bicycle ownership	Yes	51.11%	51.17%	52.44%	Unavailable	
	No	48.89%	48.83%	47.56%	Unavailable	
Transit pass holding	Yes	19.06%	19.07%	26.09%	22.67%	23.70%
	No	80.94%	80.93%	73.91%	77.33%	76.30%
Employment status	Employed full time	79.97%	79.97%	82.02%	82.95%	84.31%
	Employed part time	20.03%	20.03%	17.98%	17.05%	15.69%
Occupation	Sales and services	26.81%	26.77%	17.02%	25.60%	25.60%
	Manufacturing, construction or trades	15.08%	12.83%	10.74%	15.30%	13.04%
	General office, clerical, professional, management or technical	60.36%	58.16%	54.67%	59.00%	61.36%
Spatial controls (unweighted)			Mean	Median	Min.	Max.
Distance from home to work (km)			11.6	6	0	177.5
Distance from work to nearest sub-centre (km)			13	4	0.08	139.5
Distance from home to nearest sub-centre (km)			15	6.1	0.03	123.7
Distance from work to downtown Toronto (km)			32.3	21.7	0.1	162.4
Distance from home to downtown Toronto (km)			36.3	26.3	0.04	140.9
Job density in TAZ per square kilometre (residence)			2072.7	767.9	0.8	262619
Job density in TAZ per square kilometre (work)			16,821.5	1206.6	0.1	262619
Transit station density per square kilometre (residence)			10.8	10.9	0	60.4
Transit station density per square kilometre (work)			14.6	12.6	0	60.4

Variables are combined through question wording and/or transferrable category groupings, but the AV Survey does not capture employees’ occupation types, while the FMCS does. A multinomial logit model (available upon request) is estimated using the FMCS to predict workers’ occupation types and used to impute missing occupation data for the AV Survey. Occupation type variables take on binary one or zero for the FMCS, but the imputed values for the AV Survey are continuous based on their predicted values. This approach is considered in the weighting process.

Beyond occupation types, weights are also calculated by 21 geographic zones and based on three annual household income categories (under CA$100,000, over CA$100,000 and unknown) to retain sufficient survey observations to prioritise representativeness. The GGH is divided into 21 macro zones by aggregating functionally similar planning districts and ensuring sufficient survey observations. Weights are estimated based on place of home or place of work, each of which has 189 distinct groups of occupation type (3), household income (3) and geography (21).

Work and residential location are known at the six-digit postal code level for over 90% of respondents, and at the three-digit Forward Sortation Area level or the closest major intersection for others. Using these, spatial structure metrics are estimated in conjunction with other datasets, including job sub-centre data accessed with permission by Sweet et al. (2017), TTS data to estimate job and/or population density in the traffic analysis zone (TAZ) of a respondents’ work or residence; and General Transit Feed Specification data to measure transit services in the TAZ of a respondents’ work or residence.

### Modelling

Eight multinomial logit models are estimated: one for each of the four scenarios (before the pandemic, during the pandemic, in the autumn of 2021 and in ‘ideal’ circumstances) and each of these is estimated based on the geography of both individuals’ place of residence and their place of work. To reflect the four scenarios, respondents are asked modified versions of ‘how many days did you typically telework in a month?’ The dependent variable is treated as unordered in the multinomial logit but is in fact ordinal based on the following telework frequencies: zero days, a few days per month, one to two days per week, three to four days per week and five or more days per week. While ordered logit models could also be used, multinomial logit is preferred for two reasons. First, it can more naturally be used to translate models into market shares. Second, multinomial logit enables means of exploring non-linearities in model predictors.

Models include distance from home to work and other controls for spatial structure factors relative both to place of home and work, including distance to downtown Toronto (the major agglomeration), distance to downtown Hamilton (the other major agglomeration), distance to the nearest job sub-centre, population/job density and transit stop density. Each of these are included in initial models, but proximity to Hamilton and population density are both omitted from final models due to their statistical insignificance. Based on the existing literature, it is expected that increasing distance between home and work is likely to be associated with higher telework propensities (Moeckel, 2017), increasing distance between work and the major agglomeration (Toronto) is likely to lead to more telework (Brueckner et al., 2021; Gokan et al., 2021) and work proximity to job sub-centres is likely to be associated with lower telework propensities (Circella and Mokhtarian, 2017).

### Market estimation

Using the estimated models, weekly telework frequencies are estimated for each occupation type and geography of interest.

(1) $$P\left(Y_{i}=M\right)=\frac{e^{\beta_{\mathrm{km}}X_{\mathrm{ik}}}}{\sum_{j\in j_{m}}e^{\beta_{\mathrm{km}}X_{\mathrm{jm}}}}={\hat{Y}}_{i}$$

where *Y_i_* represents the individual *i; M* represents the possible frequencies of teleworking (never, a few days per month, one to two days per week, three to four days per week and five or more days per week); *β_km_* represents coefficients to be estimated for each telework frequency response (*m*) and independent variable (*k*); *X_ik_* represents the value of the independent variable for individual *i* and variable *k*; $j\in j_{m}$ represents option *j* within choice set *j_m_*; and *X_mk_* represents the independent variable values for variable *k* and telework frequency alternative *m*.

Equation (1) is re-estimated eight times: one for each of the four scenarios (before the pandemic, during the pandemic, in autumn 2021 and in ‘ideal’ conditions) and one each based on respondents’ places of home and work. Next, the following equation is used to estimate market shares:

(2) $${\hat{Y}}_{M}=\frac{\sum W_{i}{\hat{Y}}_{\mathrm{im}}}{\sum W_{i}},$$

where ${\hat{Y}}_{M}$ represents the mean weighted individual (*i*) predicted probability of choosing response *m*; $W_{i}{\hat{Y}}_{\mathrm{im}}$ represents the individual and occupation-specific weight for individual *i* ( $W_{i}$ ); and ${\hat{Y}}_{\mathrm{im}}$ represents the model’s predicted probability of individual *i* choosing response alternative *m*.

Equation (2) is estimated for each of the eight models for each of the 21 macro zones. Using model results, mean telework days per week are estimated using the centre points of each telework frequency category – providing indications on the geography of work and commuting considering pandemic-induced telework.

## Results

First, differences in the estimated links between built environment metrics and telework propensity are explored with respect to changes between the four model scenarios: before/during the pandemic, in the autumn of 2021 and in ‘ideal’ circumstances. Second, the market shares are mapped to illustrate the underlying changes in telework propensities.

### Model results

While models are estimated independently based on respondents’ places of residence and work, the primary results are only shown based on respondents’ place of work (see Table 2), as control variables perform almost identically. The only differences pertain to spatial structure controls (shown separately; see Table 3).

**Table 2.** Model results based on place of work (spatial controls shown separately).

Variable	Telework level	Model based on place of work (‘no teleworking’ is reference)			
		Pre-pandemic	During pandemic	Autumn 2021	Ideal
Intercept	Infrequent	−2.58***	–	–	–
	1–2×/week	−2.63***	–	–	−1.32*
	3–4×/week	−4.31***	−1.8**	−2.92***	–
	5×/week	−2**	–	−1.53**	–
Household size	Infrequent	−0.33***	−0.26***	−0.18**	–
	1–2×/week	−0.24***	−0.19**	−0.24***	–
	3–4×/week	−0.18**	–	−0.16**	–
	5×/week	–	–	–	–
Household income < CA$40,000	Infrequent	–	–	–	–
	1–2×/week	–	–	−0.47	–
	3–4×/week	–	−0.66**	–	−0.63**
	5×/week	0.47*	−0.39*	−0.37*	–
Household income > CA$100,000	Infrequent	–	–	–	–
	1–2×/week	0.44***	–	–	–
	3–4×/week	0.36*	–	–	–
	5×/week	–	0.2	–	–
Household income unknown	Infrequent	–	–	–	–
	1–2×/week	−1.03**	−0.87**	−0.7**	−0.74**
	3–4×/week	–	−0.62*	–	−0.44*
	5×/week	–	−0.46**	−0.45**	–
Household children – yes	Infrequent	0.69***	0.79***	0.48**	–
	1–2×/week	0.44**	0.66***	0.58***	–
	3–4×/week	0.54**	0.31	0.32*	–
	5×/week	–	–	–	–
Age	Infrequent	–	–	−0.01*	−0.03***
	1–2×/week	−0.01**	−0.02***	−0.02***	−0.01**
	3–4×/week	–	–	–	−0.02***
	5×/week	0.01*	–	–	–
Male – yes	Infrequent	0.41***	–	–	–
	1–2×/week	–	–	0.31**	–
	3–4×/week	–	–	–	–
	5×/week	–	−0.22**	–	−0.24**
Student – yes	Infrequent	0.96***	0.47*	0.81***	0.7**
	1–2×/week	0.88***	1.32***	0.78***	1.13***
	3–4×/week	1.33***	0.71***	1.01***	0.91***
	5×/week	–	–	–	0.41*
Education – no university	Infrequent	–	–	–	−0.48*
	1–2×/week	–	–	–	–
	3–4×/week	−0.62*	–	–	–
	5×/week	–	–	–	–
Education – graduate degree or higher	Infrequent	0.48***	0.4**	–	–
	1–2×/week	0.32*	0.61***	0.4**	0.36**
	3–4×/week	–	0.65***	0.7***	0.63***
	5×/week	–	0.65***	0.4***	0.3**
Household vehicle count	Infrequent	0.22**	0.25**	–	–
	1–2×/week	0.18*	0.21**	0.23**	–
	3–4×/week	0.25**	–	–	–
	5×/week	–	–	−0.13*	–
Bike ownership – yes	Infrequent	0.3**	0.28*	–	–
	1–2×/week	–	–	–	–
	3–4×/week	–	0.38**	0.34**	–
	5×/week	–	–	–	–
Transit pass holding – yes	Infrequent	0.46***	0.63***	–	–
	1–2×/week	0.58***	0.46**	0.38**	–
	3–4×/week	0.76***	0.45**	0.53***	–
	5×/week	–	–	–	–
Occupation – sales or services	Infrequent	–	−0.57	–	–
	1–2×/week	–	−0.69*	−0.75**	−0.74**
	3–4×/week	–	−0.98**	−0.77**	−0.82**
	5×/week	–	−1.15***	−0.81***	−0.87***
Occupation – manufacturing, construction or trades	Infrequent	–	−1.14**	–	–
	1–2×/week	−1.9***	−2.45***	−1.54***	−1.65***
	3–4×/week	−1.32**	−1.72***	−2.31***	−2.31***
	5×/week	−1.77***	−2.74***	−2.02***	−1.53***
Part-time employment status – yes	Infrequent	–	–	–	0.38*
	1–2×/week	–	–	–	–
	3–4×/week	–	–	–	−0.41**
	5×/week	–	−1.08***	−1.05***	−0.63***
Log-likelihood		−2690.3	−3129.8	−3197.3	−3586.1
McFadden –*R*-square		0.083	0.115	0.087	0.058

*Notes*: ‘–’ denotes that a variable was insignificant at the 0.10 level; significance also denoted at the 0.01 (***), 0.05 (**) and 0.10 (*) levels.

**Table 3.** Model results for spatial controls based on place of home and place of work.

Model	Variable	Telework level	Model (‘no teleworking’ is the reference level)			
			Pre-pandemic	During pandemic	Autumn 2021	Ideal
Model based on place of work	Distance from home to work (km)	Infrequent	−0.1***	−0.13***	−0.1**	–
		1–2×/week	−0.22***	−0.11**	−0.07*	–
		3–4×/week	−0.09*	–	–	–
		5×/week	−0.36***	–	−0.07**	−0.07**
	Distance work to nearest sub-centre (km)	Infrequent	–	–	−0.18	–
		1–2×/week	–	–	–	–
		3–4×/week	–	–	–	–
		5×/week	–	–	–	–
	Distance work to downtown Toronto (km)	Infrequent	–	−0.29**	–	–
		1–2×/week	–	–	–	–
		3–4×/week	–	–	–	−0.22**
		5×/week	−0.28**	−0.18**	−0.15*	−0.18*
	Distance from home to downtown Toronto (km)	Infrequent	–	–	–	–
		1–2×/week	0.4***	–	–	–
		3–4×/week	–	−0.22**	–	0.27***
		5×/week	–	–	0.24***	0.24**
	Job density in TAZ – ln	Infrequent	0.12*	−0.21**	–	0.16*
		1–2×/week	–	–	–	0.17**
		3–4×/week	–	–	0.13*	–
		5×/week	–	0.14**	0.15**	–
	Transit station density – ln	Infrequent	–	–	–	–
		1–2×/week	–	–	–	−0.12*
		3–4×/week	–	–	–	–
		5×/week	–	–	–	–
Model based on place of home	Distance from home to work (km)	Infrequent	−0.09**	−0.14***	−0.11**	–
		1–2×/week	−0.22***	−0.11**	−0.07*	–
		3–4×/week	–	–	–	–
		5×/week	−0.38***	–	−0.06*	−0.07**
	Distance home to nearest sub-centre (km)	Infrequent	−0.26***	–	–	–
		1–2×/week	–	–	–	–
		3–4×/week	–	–	–	–
		5×/week	–	–	–	–
	Distance work to downtown Toronto (km)	Infrequent	−0.17***	–	–	−0.18**
		1–2×/week	−0.29***	–	–	−0.25***
		3–4×/week	–	–	−0.15**	−0.31***
		5×/week	−0.17*	−0.33***	−0.37***	−0.28***
	Distance from home to downtown Toronto (km)	Infrequent	–	–	–	–
		1–2×/week	0.39***	–	–	0.28**
		3–4×/week	–	−0.22	–	0.25**
		5×/week	–	–	0.33***	0.25**
	Job density in TAZ – ln	Infrequent	–	–	–	–
		1–2×/week	−0.17*	–	–	–
		3–4×/week	–	–	–	–
		5×/week	–	–	–	–
	Transit station density – ln	Infrequent	–	–	–	–
		1–2×/week	0.17*	–	–	–
		3–4×/week	–	–	–	0.18**
		5×/week	–	–	–	–

*Notes*: ‘–’ denotes that a variable was insignificant at the 0.10 level; significance also denoted at the 0.01 (***), 0.05 (**) and 0.10 (*) levels.

The results support many findings from the existing research. Both larger households and households with children are associated with lower probabilities of teleworking at intermediate frequencies (but not in the ‘ideal’ scenario). Findings on household income are highly inconsistent. In contrast, having a graduate degree (perhaps a better indicator of job type) is highly significantly associated with telework – particularly during and after the pandemic.

Each of the mobility controls are significant before/during the pandemic and in the autumn of 2021, but none are significant in the ‘ideal’ telework model. Having more household vehicles, holding a transit pass and owning a bicycle are associated with higher intermediate rates of teleworking (occasionally). This suggests that telework is a general time and resource adaptation strategy in all scenarios except the ‘ideal’ conditions – implying that ‘ideal’ is a matter of personal choice. This supports the hypothesis of Shearmur (2021) that with more complex work at home arrangements, the strength and importance of trade-offs embedded in bid rent theory may need to be revisited.

Built environment controls indicate two consistent findings. First, contrary to many previous studies, teleworking and longer work–home disconnects are not linked. Instead, longer work–home disconnect is associated with lower intermediate frequency teleworking in all scenarios other than ‘ideal’. Moreover, longer work–home disconnect is associated with lower probabilities of teleworking five days a week (except during the pandemic).

Second, findings indicate a poor outlook for city-regions to retain traditional monocentric urban agglomeration economies. But there is also no evidence that proximity to sub-centres is linked with teleworking. The most important controls relate to distance from home or work to downtown Toronto: the further one lives or works from Toronto, the less likely one is to telework.

### Market estimation

This study further estimates typical teleworking (mean teleworking days per worker) in each of the 21 macro zones. Weekly telework ranges from 0.59 to 0.92 days before the pandemic, while it ranges between 1.34 and 3.25 days during the pandemic (see Table 4). Comparing pre-pandemic with ‘ideal’ teleworking, employees’‘ideals’ represent one additional teleworking day per week.

**Table 4.** Market estimates: workers’ mean weekly telework frequencies.

Zone #	Macro zone name	Based on place of work					Based on place of home				
		Pre-pandemic	During pandemic	Autumn 2021	Ideal	Change (pre–ideal)	Pre-pandemic	During pandemic	Autumn 2021	Ideal	Change (pre–ideal)
Total		0.75	2.01	1.75	1.75	+1.0	0.75	1.96	1.7	1.73	+0.98
1	Downtown Toronto	0.86	3.25	2.79	2.33	1.47	0.92	2.74	2.11	1.89	0.97
2	Midtown Toronto	0.81	2.56	2.16	2	1.19	0.89	2.69	2.25	1.95	1.06
3	North York (Toronto)	0.74	2.23	1.9	1.85	1.11	0.79	2.32	2.03	1.88	1.09
4	Etobicoke (Toronto)	0.78	2.05	1.7	1.75	0.97	0.8	2.23	1.92	1.84	1.04
5	Scarborough (Toronto)	0.83	1.89	1.67	1.74	0.91	0.83	2.12	1.89	1.86	1.03
6	West-End (Toronto)	0.92	2.06	1.73	1.83	0.91	0.87	2.29	1.89	1.84	0.97
7	East End / York (Toronto)	0.83	2.15	1.82	1.87	1.04	0.84	2.49	2.12	1.96	1.12
8	Durham region	0.75	1.53	1.32	1.51	0.76	0.71	1.89	1.61	1.75	1.04
9	York region (excluding Vaughan and Markham)	0.69	1.62	1.41	1.55	0.86	0.7	1.95	1.68	1.71	1.01
10	City of Markham	0.71	2	1.72	1.73	1.02	0.69	2.13	1.83	1.83	1.14
11	City of Vaughan	0.67	1.87	1.55	1.62	0.95	0.74	2.03	1.71	1.72	0.98
12	Town of Caledon and city of Brampton	0.8	1.55	1.38	1.56	0.76	0.78	1.81	1.62	1.74	0.96
13	City of Mississauga	0.71	1.94	1.7	1.74	1.03	0.72	1.96	1.75	1.75	1.03
14	Halton region	0.68	1.59	1.44	1.53	0.85	0.71	2.02	1.77	1.72	1.01
15	Hamilton region	0.59	1.42	1.22	1.41	0.82	0.62	1.49	1.28	1.41	0.79
16	Hamilton city	0.72	1.63	1.55	1.63	0.91	0.62	1.48	1.42	1.51	0.89
17	Niagara region	0.72	1.36	1.18	1.43	0.71	0.73	1.49	1.3	1.59	0.86
18	Waterloo region	0.68	1.68	1.62	1.67	0.99	0.68	1.61	1.55	1.61	0.93
19	City of Guelph and Wellington region	0.68	1.6	1.4	1.57	0.89	0.7	1.75	1.51	1.65	0.95
20	City of Barrie and regions of Dufferin and Simcoe	0.67	1.34	1.14	1.36	0.69	0.65	1.44	1.23	1.45	0.8
21	Peterborough city and region	0.62	1.55	1.35	1.49	0.87	0.65	1.56	1.3	1.56	0.91

**Figure 1.**
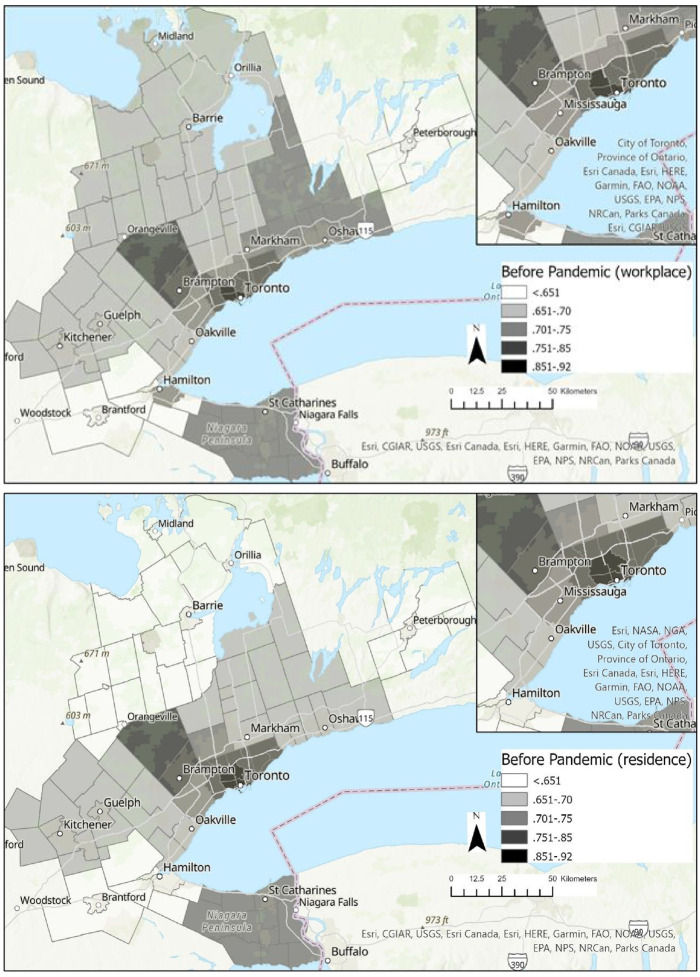
Pre-pandemic mean weekly telework days based on place of work (top) and residence (bottom).

**Figure 2.**
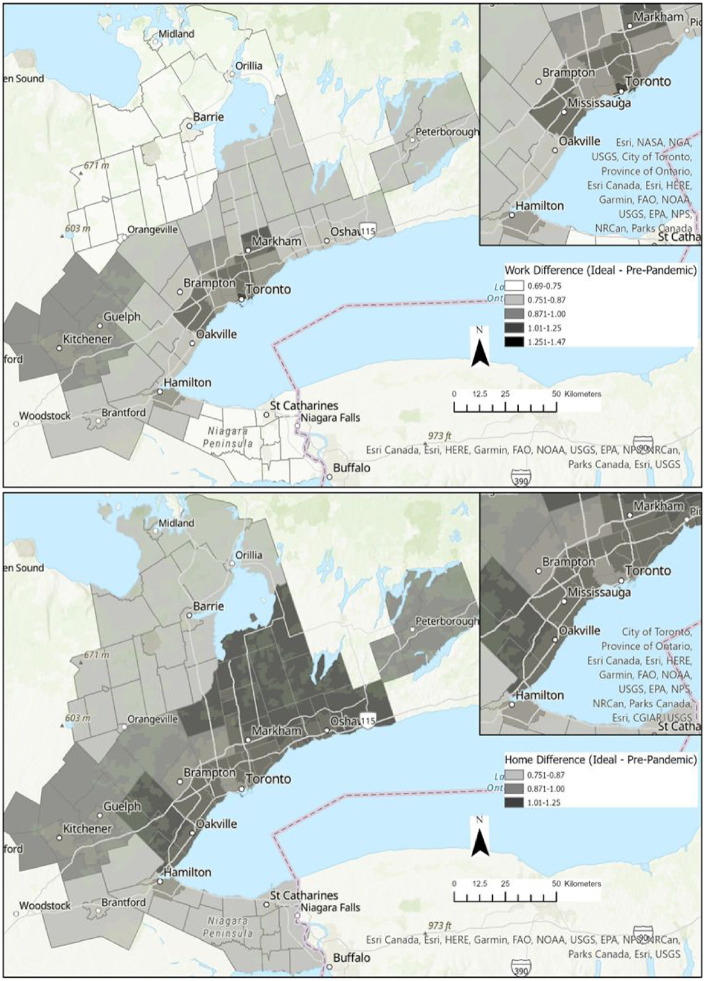
Weekly increase in telework days between pre-pandemic and ‘ideal’ based on place of work (top) and home (bottom).

While pre-pandemic telework was already higher in Toronto (Figure 1), telework growth during the pandemic was more clustered around downtown Toronto based on work location than based on residential location (which is more equally distributed). Teleworking changes between pre-pandemic and ‘ideal’ circumstances indicate significant teleworking growth based on work locations concentrated on downtown Toronto, but this is more equally distributed based on residential location (Figure 2). To illustrate the difference, Gini coefficients were calculated with respect to the weighted sample-adjusted differences between pre-pandemic and ‘ideal’ teleworking for the 21 macro zones. The Gini coefficients were 0.22 based on place of work and 0.12 based on place of residence – suggesting an 83% difference in the inequality of how additional teleworking may manifest spatially. Estimates are further transformed to calculate changes in daytime population using the difference between telework growth based on residential and job locations. Only downtown Toronto is expected to have a significant decrease in daytime population (−27%) while no other zone is expected to have more than a 4% decrease. Nevertheless, the construct of daytime population should be interpreted carefully, given the different broader activities and roles of employees at their residence compared with at a formal workplace. Select zones are anticipated to have net increases, including a mix of suburbs/exurbs, Durham (+20%), Caledon and Brampton (+16%) and the ex-urban York Region (+13%); and highly urbanised Toronto neighbourhoods such as the West-End (+17%), East End/York (+14%) and Scarborough (+12%); see online Appendix.

## Conclusion

The Covid-19 pandemic dramatically increased teleworking. But it is unclear whether such high levels of teleworking are likely to continue in the future and how they will change urban spatial structure. Using survey data from autumn 2021, this study addresses these questions using models to explore the changing spatial geography of telework. If even a small share of the current and possible (workers’ stated ‘ideal’) teleworking rates are realised, the geographies of work will be dramatically impacted. Urban economic theory commonly suggests that telework is likely to disconnect work and home and to induce residential sprawl, but this study suggests an even more dramatic portrait. Working closer to Toronto is associated with more teleworking, but longer work–home disconnects are not, suggesting that teleworking is not featuring in expected commute–location trade-offs. In short, telework appears to be weakening bid rent theory and supports a more complex narrative of places of work, as anticipated by Shearmur (2021). Job sub-centring, population and/or job density, and transit service density likewise play minor roles compared with where workers live and work relative to downtown Toronto. The geography of telework (and changes thereof) is much more concentrated with respect to the work locations, rather than residential locations. In terms of changes in daytime working populations, the outlook for downtown Toronto is poor, while most other neighbourhoods can expect net stability or increases. But caution is also warranted on this finding, as it is unclear how commercial real estate operators will respond and whether changes in aggregate economic activity (not just redistributed activity) may emerge.

Ironically, findings suggest that in-person work is likely to be reduced in precisely those places that have been designed for proximate interaction – notably downtown Toronto. Given the high rents and advantages in such places, one might expect firms to reprogramme how office space is used – leading to differences between the geographies of collaborative, rather than task-based work. Bid rent theory’s core assumption – that access to the central market is desired – is under pressure. Regional labour markets may very well be the anchoring scale of urban agglomeration, but findings from this study suggest significant change is likely for local place-based agglomerations, like downtowns. This begs the question whether knowledge-based agglomeration benefits accrue through regular engagement, random interactions or non-random programmed interactions.

Caution is warranted given the uncertainty in future teleworking. This study’s findings should be interpreted as latent tendencies towards change, rather than as known futures. Likewise, given that findings represent employees’ perceptions (which are affected by firms’ initiatives to entice employees back to work), they appear to represent upper bounds to future teleworking. Regardless, planners should explore long-term planning interventions contingently and examine the robustness of projects considering multiple possible scenarios, including teleworking. For transportation planners, findings from this study suggest that land markets and commuting (and hence the valuation thereof) may be in the process of undergoing important changes which warrant re-evaluation in planning processes and economic business cases.

## Supplemental Material

sj-docx-1-usj-10.1177_00420980231186499 – Supplemental material for What might working from home mean for the geography of work and commuting in the Greater Golden Horseshoe, Canada? Click here for additional data file. Supplemental material, sj-docx-1-usj-10.1177_00420980231186499 for What might working from home mean for the geography of work and commuting in the Greater Golden Horseshoe, Canada? by Matthias Sweet and Darren M Scott in Urban Studies
