# Supplementary material for: What might working from home mean for the geography of work and commuting in the Greater Golden Horseshoe, Canada?
Source: Urban Stud. 2023 Aug 7;61(3):567–88. doi: 10.1177/00420980231186499 (PMC10830397; doi:10.1177/00420980231186499)
Supplement: sj-docx-1-usj-10.1177_00420980231186499 – Supplemental material for What might working from home mean for the geography of work and commuting in the Greater Golden Horseshoe, Canada? [file sj-docx-1-usj-10.1177_00420980231186499.docx]

**Online Appendix.**


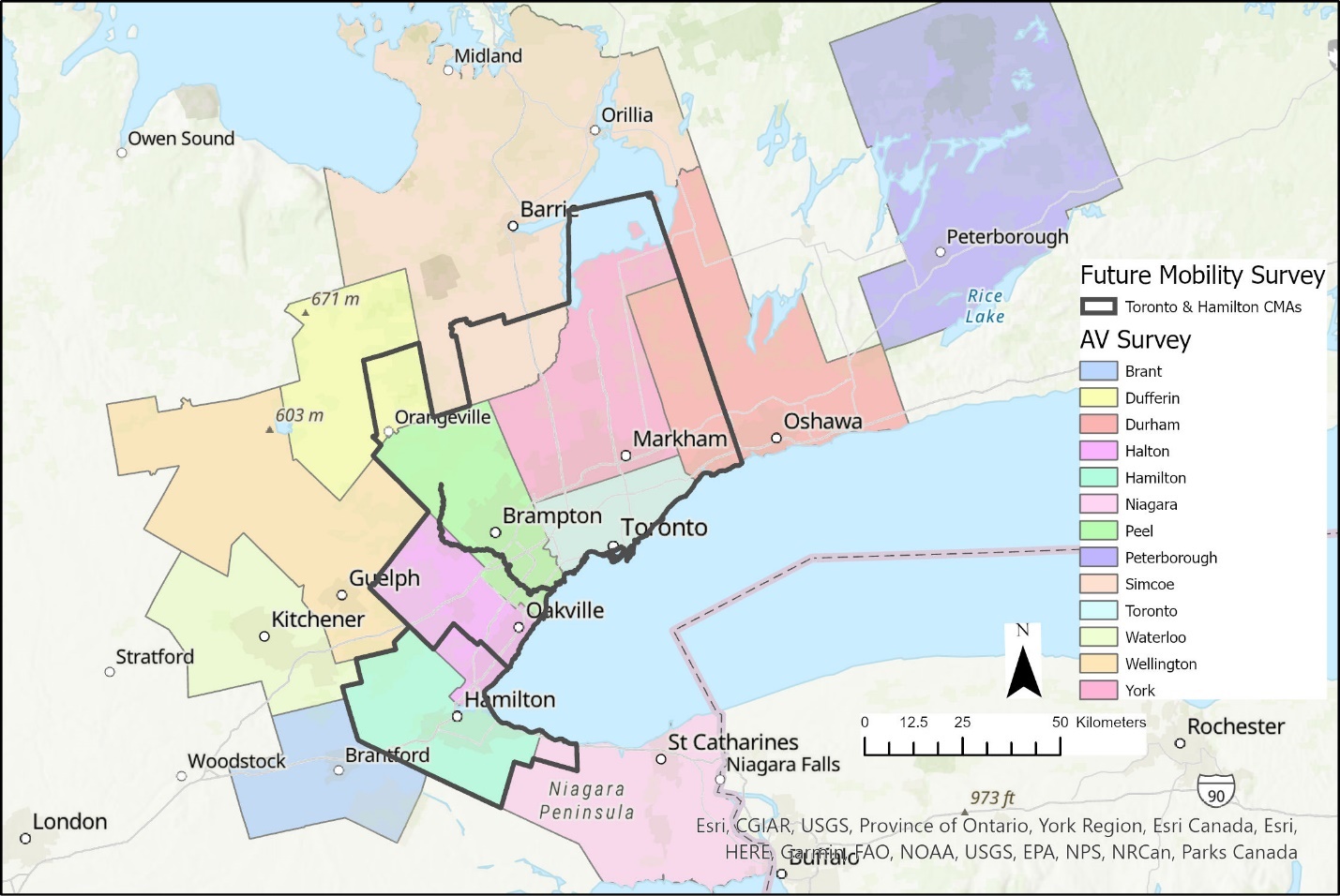


Figure A.1. Greater Golden Horseshoe - Future Mobility in Canada Survey and AV Survey Geographies


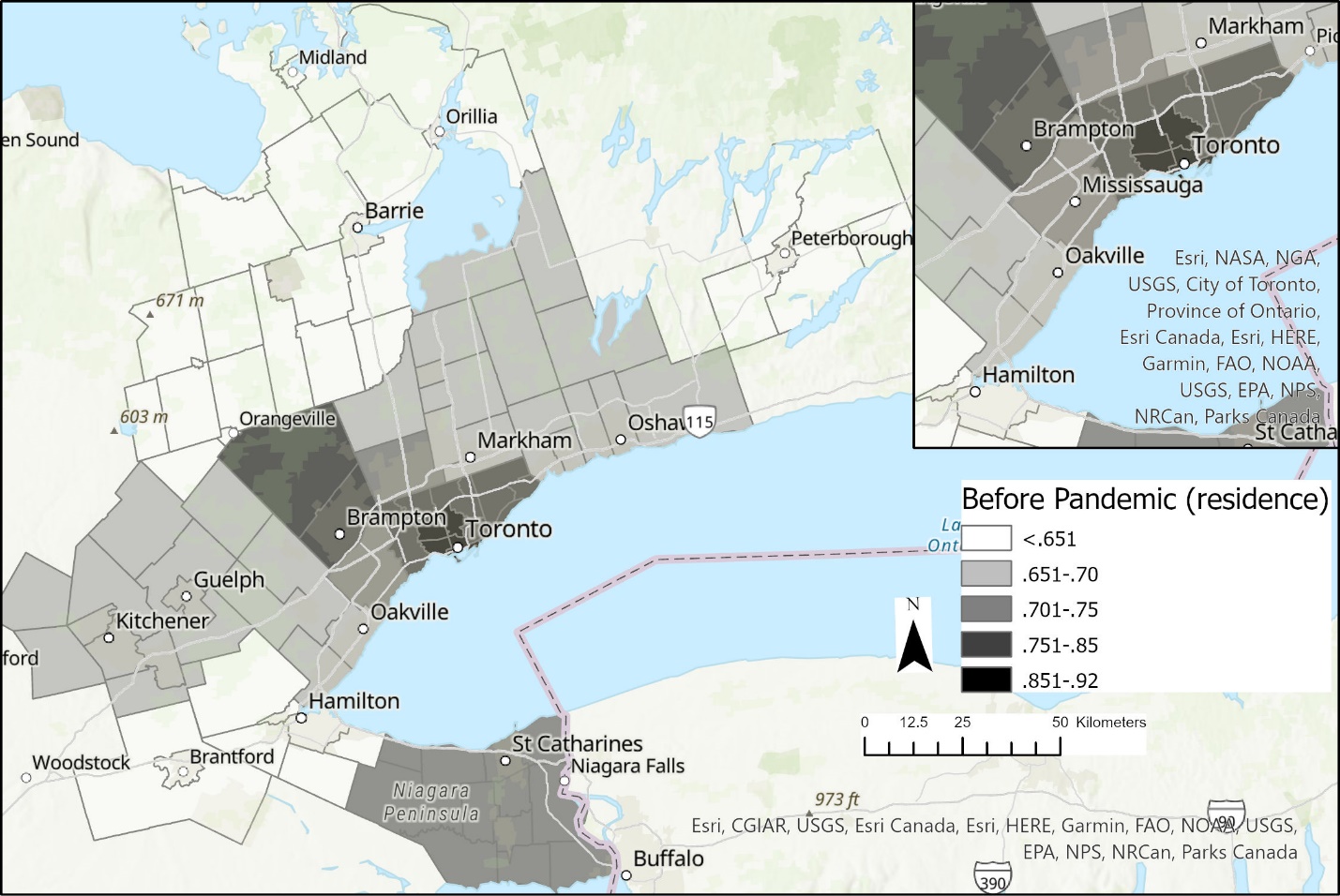


Figure A.2. Pre-Pandemic Weekly Teleworking (days per week) Based on Place of Residence


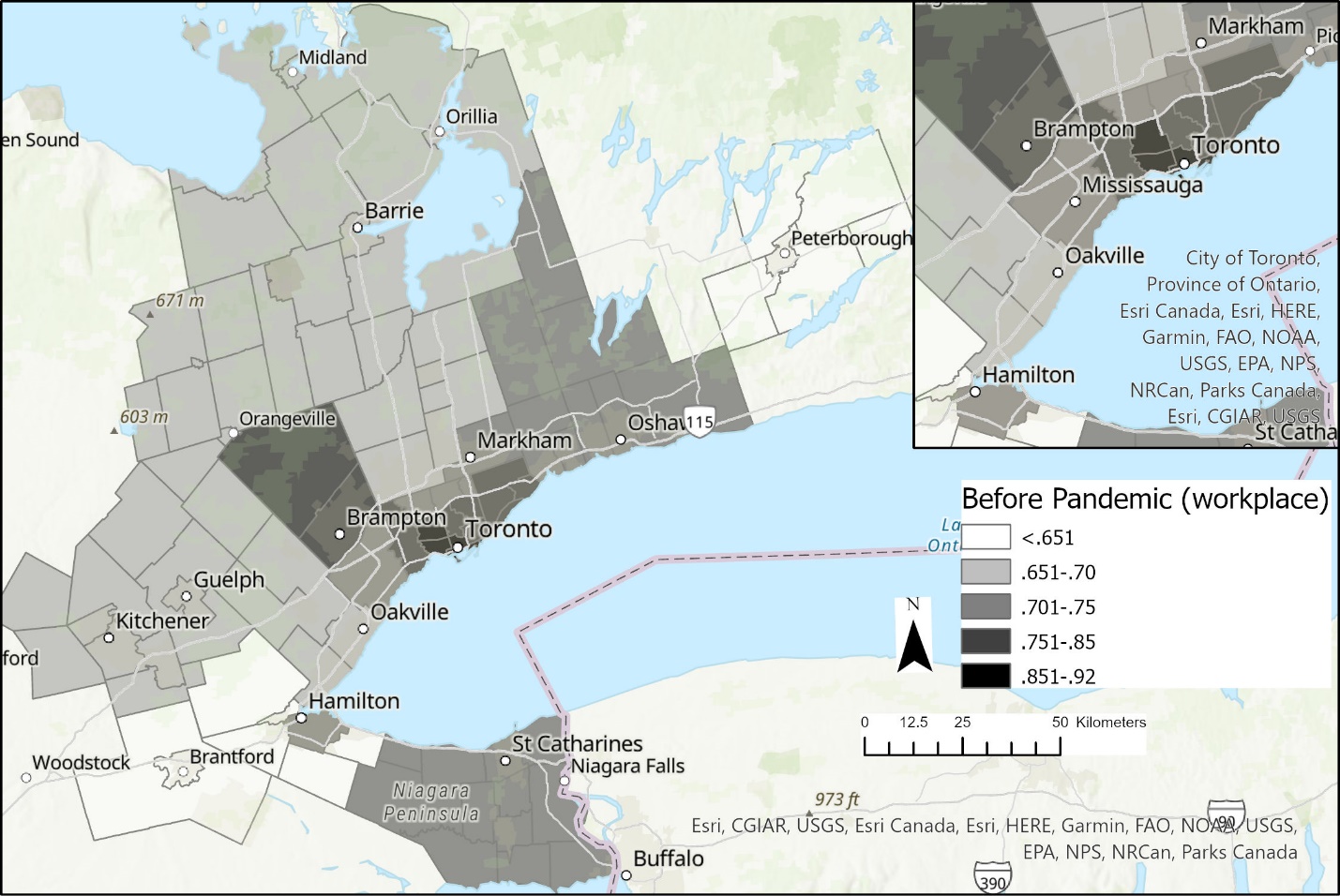


Figure A.3. Pre-Pandemic Weekly Teleworking (days per week) Based on Place of Work


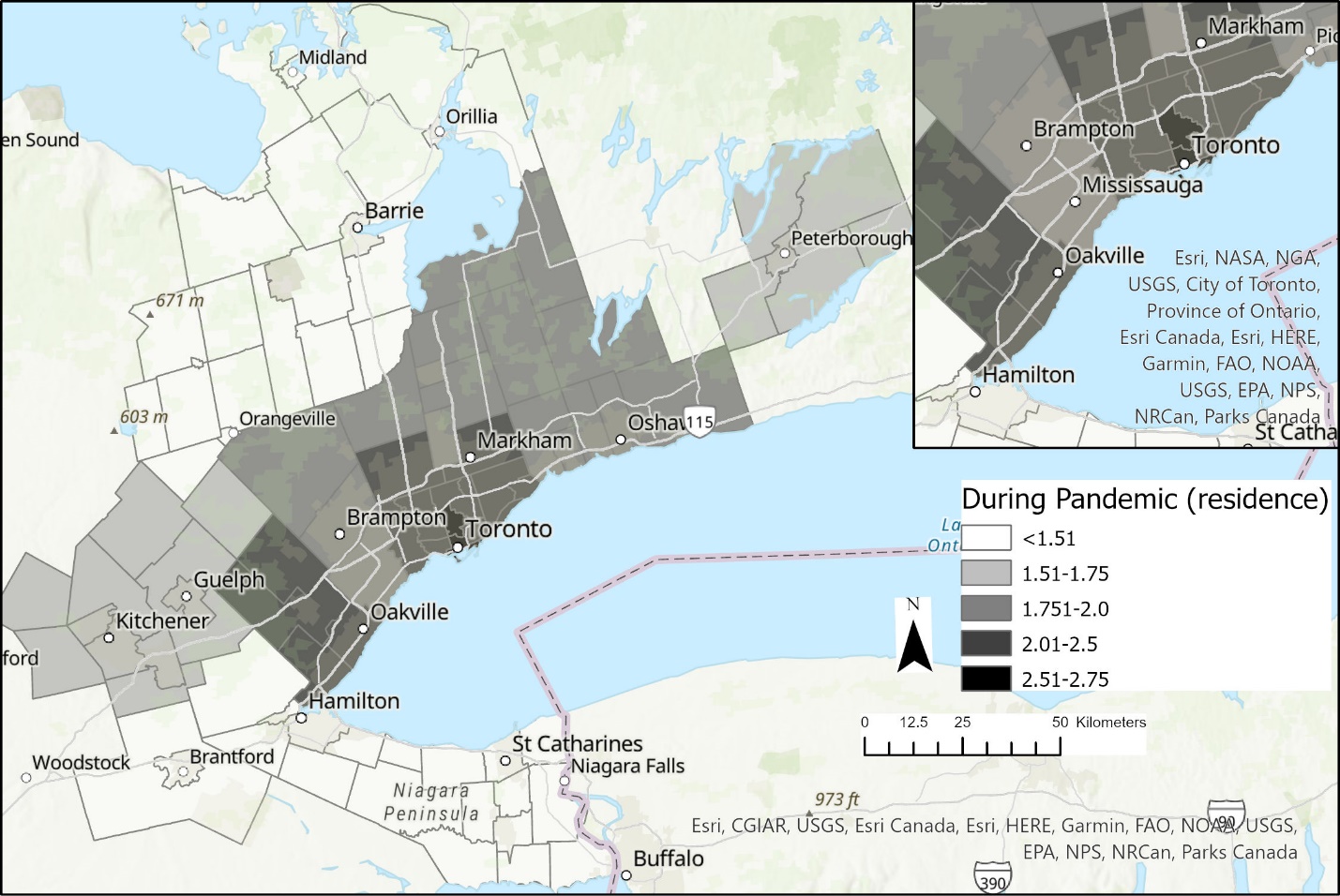


Figure A.4. During Pandemic Weekly Teleworking (days per week) Based on Place of Residence


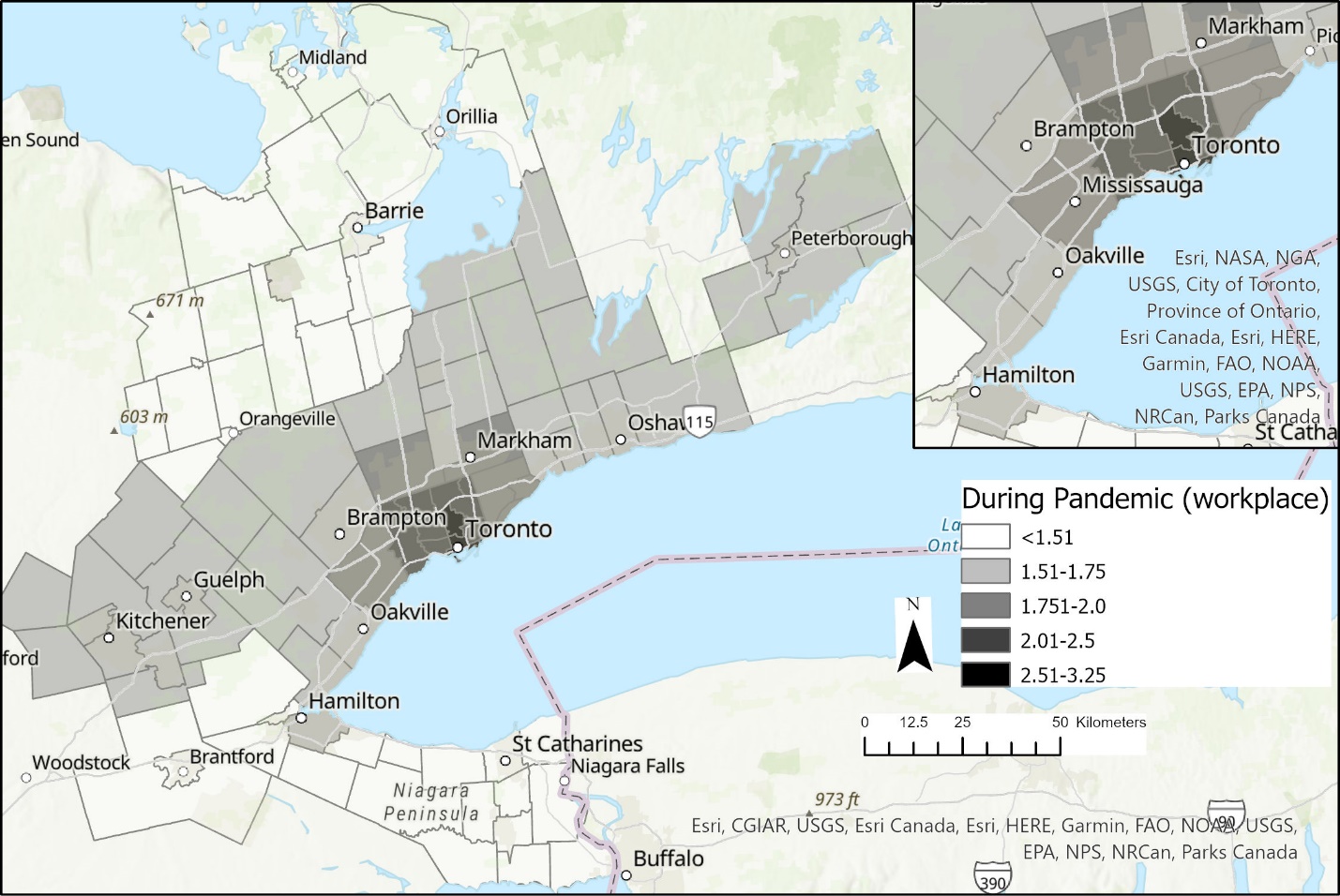


Figure A.5. During Pandemic Weekly Teleworking (days per week) Based on Place of Work


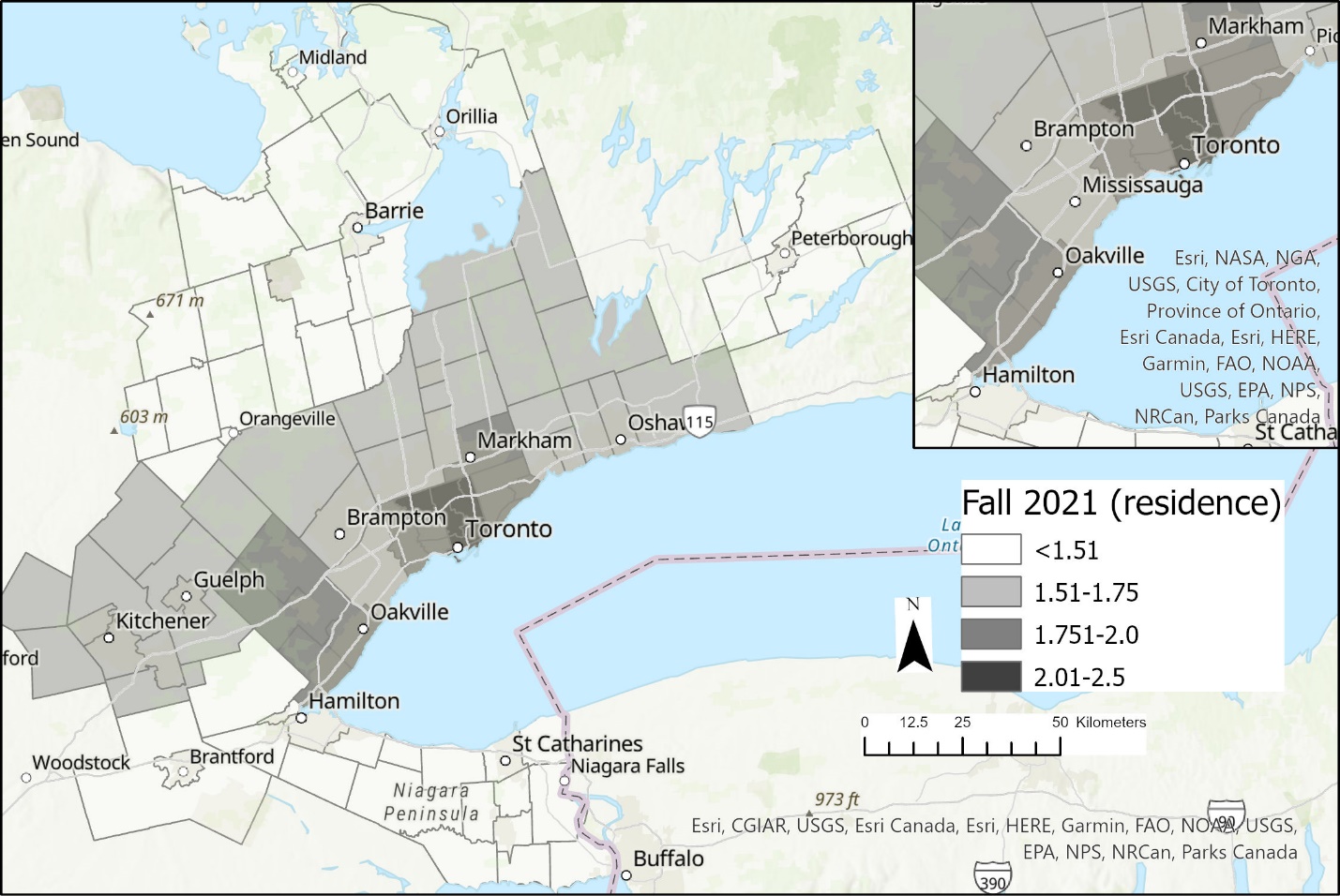


Figure A.6. Fall 2021 Weekly Teleworking (days per week) Based on Place of Residence


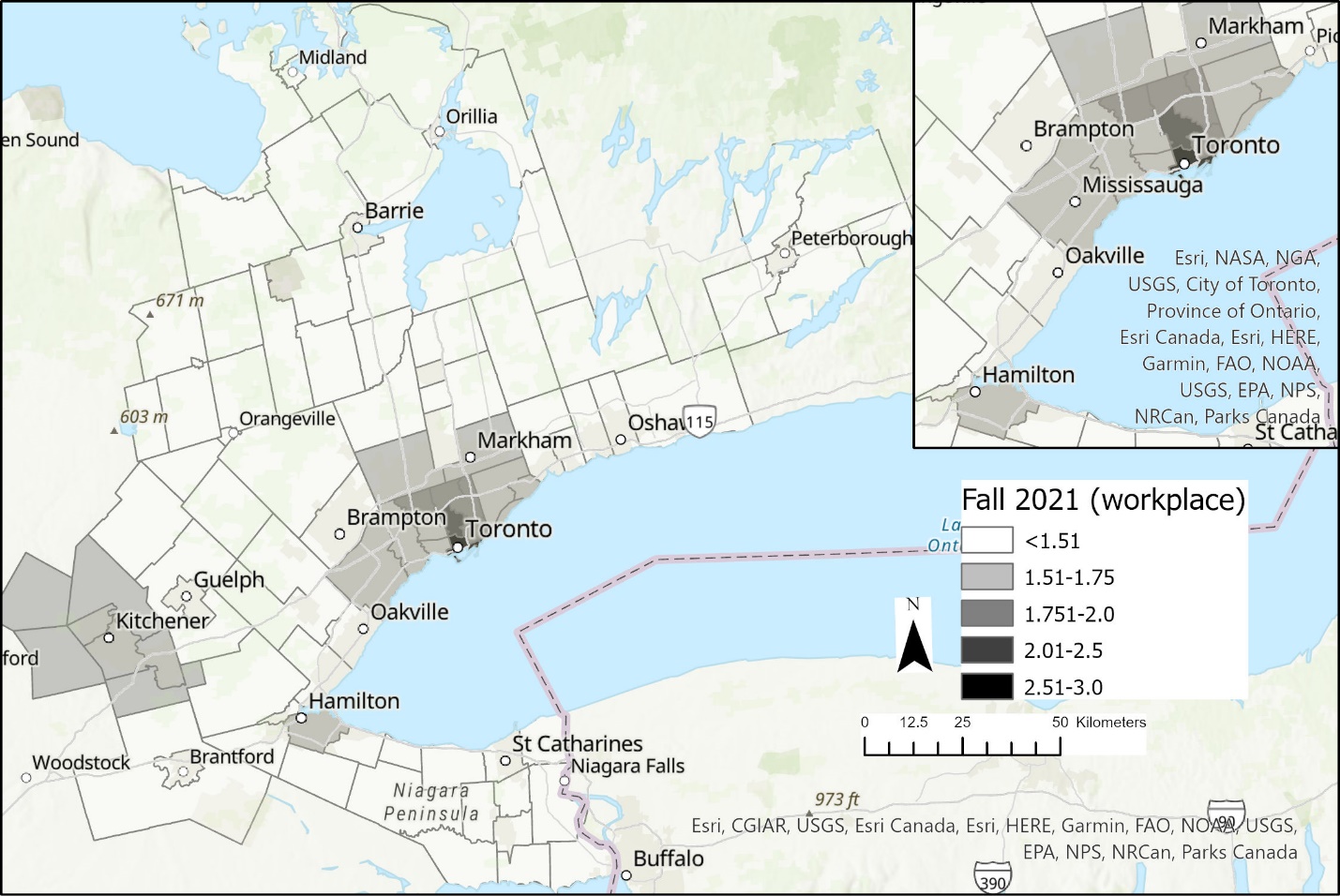


Figure A.7. Fall 2021 Weekly Teleworking (days per week) Based on Place of Work


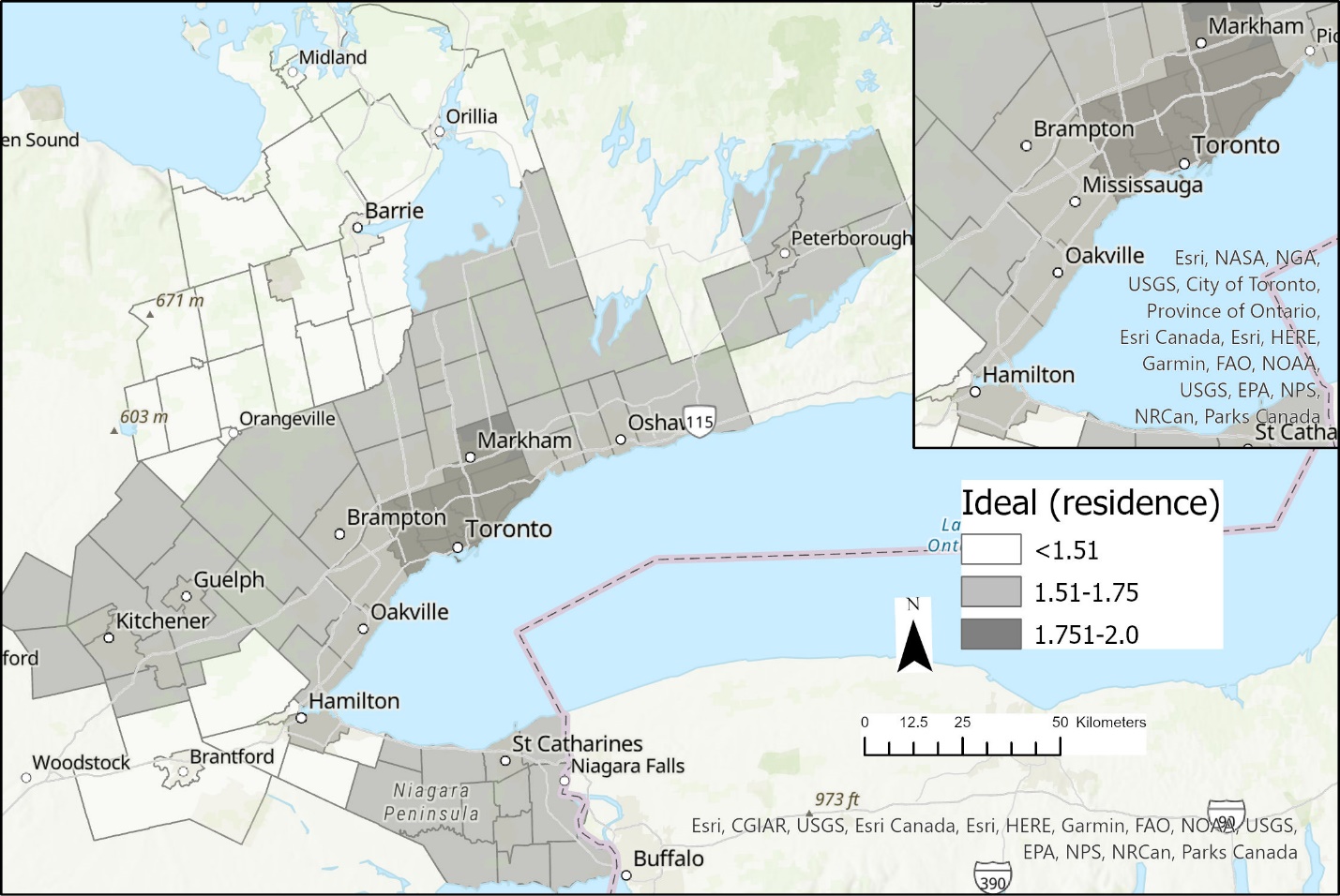


Figure A.8. Ideal Weekly Teleworking (days per week) Based on Place of Residence


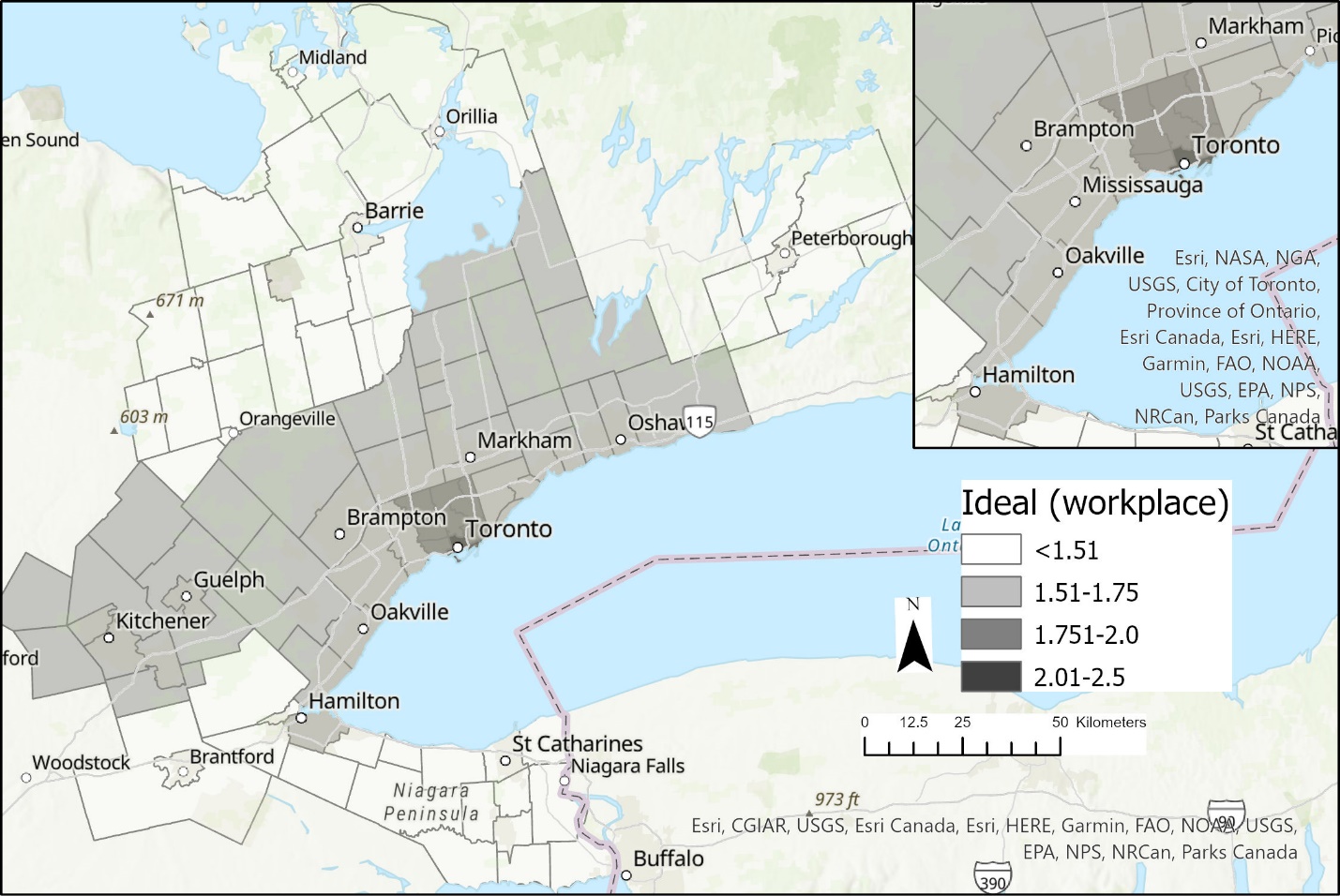


Figure A.9. Ideal Weekly Teleworking (days per week) Based on Place of Work


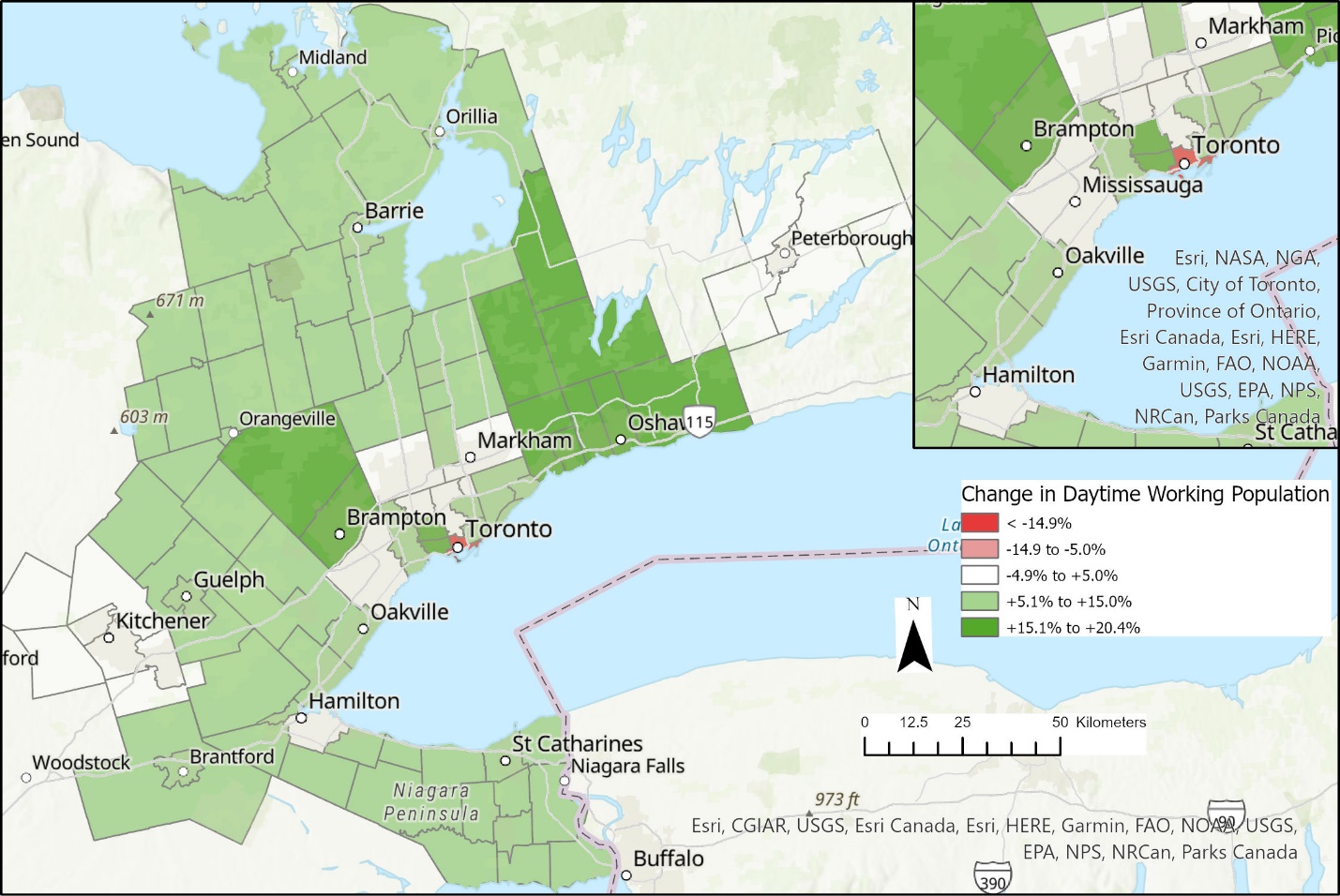


Figure A.10. Estimated Change (%) in Daytime Working Population (Teleworkers and Non-Teleworkers)
